# Resolutions of the Erie Co. Mod. Society, Relative to Colleges Conferring Degrees upon Students Who Have Pursued Their Medical Studies with Irregular Practitioners

**Published:** 1846-07

**Authors:** 


*Resolutions of the Erie Co. Med. Society, relative to Colleges conferring degrees upon students who have pursued their medical studies with irregular practitioners.*

The following resolutions were adopted by the Medical Society of this county at its late meeting, and directed to be published in this journal.

*Whereas*, it has appeared to this Society, that certain Colleges in this state have conferred degrees upon students who had pursued their studies with an irregular practitioner; and, whereas, a diploma conferred under such circumstances is in direct violation of law, calculated to encourage

Quackery, remove the dividing line between the regular and the irregular practitioner, and lower the standard of medical education: therefore,

1. *Resolved*, That, in the opinion of this Society, the graduation of a pupil by any medical institution, without proper efforts to ascertain whether he has complied with the regulations provided by law, in pursuing his studies, for a full period, with a regular physician, in good standing; and more especially, accepting a certificate from an irregular practitioner, deserve the strongest reprobation of the medical profession.

2. *Resolved*, That it is the duty of medical practitioners who have at heart the welfare and honor of the profession, to discountenance institutions who are obnoxious to these charges.

3. *Resolved*, That the Secretary be directed to transmit these resolutions and preamble to all the Medical Colleges of this state, to the State Medical Society, and to the Editor of the Buffalo Medical Journal, for publication.

